# Cytochrome P450s Are Essential for Insecticide Tolerance in the Endoparasitoid Wasp *Meteorus pulchricornis* (Hymenoptera: Braconidae)

**DOI:** 10.3390/insects12070651

**Published:** 2021-07-16

**Authors:** Xiaorong Xing, Mengwen Yan, Huilin Pang, Fu’an Wu, Jun Wang, Sheng Sheng

**Affiliations:** 1 Jiangsu Key Laboratory of Sericultural Biology and Biotechnology, School of Biotechnology, Jiangsu University of Science and Technology, Zhenjiang 212018, China; 172211801110@stu.just.edu.cn (X.X.); 182211802111@stu.just.edu.cn (M.Y.); 182211803105@stu.just.edu.cn (H.P.); 198700001882@just.edu.cn (F.W.); wangjun@just.edu.cn (J.W.); 2 The Key Laboratory of Silkworm and Mulberry Genetic Improvement, Ministry of Agriculture, Sericultural Research Institute, Chinese Academy of Agricultural Science, Zhenjiang 212018, China

**Keywords:** *Meteorus pulchricornis*, cytochrome P450s, detoxification, insecticide tolerance

## Abstract

**Simple Summary:**

P450s are involved in the detoxification process in insects; however, less attention has been paid for their roles in parasitoid wasps. Here, we identified 28 P450s from the previous constructed transcriptome dataset of *Meteorus pulchricornis* and validated their expression patterns under insecticides exposure. Furthermore, we silenced *CYP369B3* in adult wasps and demonstrated that the knocking down *CYP369B3* significantly increased of the mortality of adult wasps when they were exposed to each chemical insecticide, phoxim, cypermethrin, and chlorfenapyr, respectively. The present study provides a theoretical base for functional research on P450 genes in parasitoid wasps.

**Abstract:**

With the widespread application of insecticides, parasitoid wasps may also be under risk when exposed to insecticides directly at their free-living stages. The endoparasitoid wasp *Meteorus pulchricornis* is the predominant natural enemy of many lepidopteran pests, such as *Spodoptera litura* and *Helicoverpa armigera*. The cytochrome P450 monooxygenases constitute a ubiquitous and complex superfamily of hydrophobic, haem-containing enzymes. P450s are involved in the detoxification of many xenobiotics. However, their exact roles in the tolerance mechanism in parasitoids toward insecticides has received less attention. Here, 28 P450 genes in *M. pulchricornis* were identified from a previously constructed transcriptome dataset. These P450 genes belonged to CYP2, -3, and -4, and mitochondrial clans. Subsequently, eight candidate *MpulCYPs* were selected from four CYP clans to validate their expression patterns under phoxim, cypermethrin, and chlorfenapyr exposure by qRT-PCR. The results showed that all three insecticides had significant effects on the expression of *MpulCYPs*. To further study the function of P450s, *CYP369B3* was silenced, and its expression levels of *CYP369B3* were significantly decreased. Survival analysis indicated that after dsRNA injection, the mortality rate of wasps was significantly increased when *M. pulchricornis* females were exposed to insecticides compared to control groups. Our findings provide a theoretical base for elucidating the mechanism of insecticide tolerance and promote functional research on P450 genes in parasitoid wasps.

## 1. Introduction

Mulberry foliage is the exclusive food resource of the silkworm *Bombyx mori* (Lepidoptera: Bombycidae) and is easily damaged by mulberry pests, resulting in a great threat for the sericulture industry. Among these, the common cutworm *Spodoptera litura* (Lepidoptera: Noctuidae) has destroyed mulberry much more severely in recent years in China. Therefore, effective control of this pest is vital. Traditional chemical control often fails due to the lack of novel effective insecticides and high resistance towards commonly used insecticides in *S. litura*. Alternatively, other control methods are urgently needed. For decades, biological control agents, such as parasitoid wasps, have been shown to suppress the population of *S. litura* successfully. In mulberry fields, many parasitoid wasps of *S. litura* have been investigated, and attempts to use them to control *S. litura* have also been promoted.

An effective integrated pest management (IPM) practice is based on balancing the use of chemical insecticides and parasitoid wasps, aiming to create a favorable condition for biological agents. It is well accepted that chemical insecticides usually pose a great challenge for parasitoids not only for survival but also for parasitism factors, such as the parasitism rate or foraging behavior. In other words, releasing parasitoid wasps depends on a clear understanding of their physiology under the pressure of insecticides. Detoxification enzymes are essential for the survival of insects, including parasitoid wasps, when they are exposed to insecticides. In insects, cytochrome P450 monooxygenases (P450s) have received considerable attention for their roles in the detoxification of insecticides, plant secondary metabolites, and toxic pollutants [1]. Cytochrome P450s, also known as mixed function oxidases (MFO), are terminal oxidases in the cytochrome P450 enzyme family. The structure of cytochrome P450s contain nearly 13 α-helices and 4 β-sheets, among which the highly conserved core region includes haem-binding motifs (FxxGxxxCxG), PERF (PxxFxPE/DRF), Helix-I (A/GGxD/ETT/S), Helix-K (ExxR), and Helix-C (WxxxR) [2]. Based on their phylogenetic classification, insect P450s can be divided into four major clans: CYP2, CYP3, CYP4, and mitochondrial clans. In insects, P450s are also involved in the biosynthetic and catabolic processes of juvenile hormones, ecdysterone, and cuticular hydrocarbon, which regulate insect growth and development [3].

Determining the gene expression level under insecticide stress is a common method to evaluate whether detoxification enzymes are involved in insect resistance [4,5]. Exposure to an LD_5_ dose of lambda-cyhalothrin significantly upregulated five P450 genes (*CYP4M59, CYP6AE119, CYP6AE120, CYP6AE121,* and *CYP6BD18*) in *Pieris rapae* larvae [6]. λ-Cyhalothrin exposure enhanced P450 activity and induced the expression of *CYP6AB12* significantly in *S. litura* larvae [7]. The expression level of P450s in *Bemisia tabaci* with resistance to neonicotinoid insecticides was higher than that of sensitive strains [8]. However, compared to insect pests, fewer studies have been conducted to elucidate the sensitivity and detoxification mechanisms of parasitoid wasps toward insecticides.

In mulberry fields, phoxim (benzoyl cyanide-*O*-(diethoxyphosphinothioyl)oxime), cypermethrin (cyano-3-phenoxybenzyl-2,2-dimethyl-3-(2,2-dichlorovinyl)cyclopropanecarboxylate), and chlorfenapyr (4-bromo-2-(4-chlorophenyl)-1-ethoxymethyl-5-trifluoromethylpyrrole-3-carbonitrile) are commonly used insecticides in chemical pest control [9]. The solitary endoparasitoid *M. pulchricornis* (Hymenoptera: Braconidae) is the predominant natural enemy of many lepidopteran pests, such as *S. litura*, *Helicoverpa armigera*, and *Lymantria dispar.* It has been reported that *S. litura* can be attacked by *M. pulchricornis* at a rate of 20–30% in soybean fields [10]. Previous studies demonstrated that glutathione s-transferase genes were upregulated after *M. pulchricornis* females were exposed to commonly used insecticides in mulberry fields, such as phoxim and cypermethrin [11]. However, the roles of P450s in *M. pulchricornis* when they are under insecticide stress remain unknown.

The present study aimed to identify the candidate P450 genes in *M. pulchricornis* and validate their expression levels when female wasps were treated with commonly used insecticides in mulberry. Furthermore, RNA interference was performed to investigate the function of P450s by silencing the expression of selected P450s genes. The information gained from this study can improve the understanding of the detoxification mechanism of *M. pulchricornis* and provide a reference for the protection of parasitoid wasps.

## 2. Materials and Methods

### 2.1. Insects

Since *M. pulchricornis* is thelytokous, all stocks of this parasitoid are female [12,13]. Parasitoid adults were reared on the larvae of *S. litura*, which were collected from a mulberry field in Jiangsu University of Science and Technology (Zhenjiang, China). The adult parasitoid wasps were fed a 10% (*w*/*w*) honey solution as a supplement in glass culture tubes (2.2-cm diameter, 8-cm height, plugged with gauze). *S. litura* larvae were fed an artificial diet in plastic cases (13-cm length, 13-cm width, and 6-cm height) [14], and the adults were furnished with strips of paper as a substrate for oviposition in organza-covered cages with a 10% honey solution (20-cm length, 20-cm width, and 30-cm height) [10]. Round sheets of mulberry leaves (6.5-cm diameter) were placed in plastic boxes (6.5-cm diameter) with punched lids and lined with agar gel (3%). Each box contained twenty third-instar *S. litura* larvae and two fifteen-day-old wasps for parasitism. Insects were reared after 8 h of parasitism. The rearing conditions of both insects were 25 ± 1 °C, 60–80% RH, and a photoperiod of 14:10 h (L:D).

### 2.2. Identification of P450 Genes in M. pulchricornis

P450 genes were identified from the previously constructed transcriptome database of *M. pulchricornis* (GenBank accession number: *SRR8981255*; [11]). The tBLASTn algorithm (E value < 10^−5^) was used to identify candidate unigenes encoding putative P450s in *M. pulchricornis* against the reference sequences of *Apis mellifera*, *Drosophila melanogaster, Nasonia vitripennis,* and *S. litura*. Putative genes were checked manually using the BlastX program by the nr database to confirm their identity with other insect P450 genes. According to the conservative motif of insects, the genes with the characteristic signal sequence of insect P450s were identified as the candidate P450 genes in *M. pulchricorni*. All identified P450 genes were named and classified by the Cytochrome P450 Nomenclature Committee.

### 2.3. Sequence and Phylogenetic Analyses

The ORF Finder (https://www.ncbi.nlm.nih.gov/orffinder/, accessed on 18 October 2020) was used to predict the open reading frames (ORFs) of putative *P450* genes, and ExPASy (www.expasy.org/tools/protparam.html, accessed on 18 October 2020) was used to predict the molecular weight and theoretical isoelectric point (pI). SignalP (https://www.ncbi.nlm.nih.gov/Structure/bwrpsb/bwrpsb.cgi, accessed on 18 October 2020) was used to predict the N-terminal signal peptide and the CD-Search tool (https://www.ncbi.nlm.nih.gov/Structure/bwrpsb/bwrpsb.cgi, accessed on 18 October 2020) to predict conservative domains. A phylogenetic tree was constructed by MEGA7.0 software using the neighbor-joining method with 1000 bootstrap replications [15] and decorated by FigTree1.43.

### 2.4. Insecticide Treatment

Phoxim (purity, 99%) and cypermethrin (purity, 99.7%) analytical standards were purchased from Aladdin (Shanghai, China). Chlorfenapyr (purity, 99%) was obtained from Nanjing Agricultural University, China. According to our previous results, the LC_10_ of phoxim and cypermethrin was 0.1 and 2.0 mg/L, respectively [16], and these two chemicals were dissolved in analytical-grade acetone to prepare working solution. Due to chlorfenapyr being newly promoted in the mulberry fields for controlling the pests, no sublethal dose under indoor condition can be acquired. Therefore, the appropriately 10% field application dose (4 mg/L) [17] was prepared to obtain the chlorfenapyr working solution which was also dissolved in acetone. The tested wasps were originally kept in glass tubes (2.2-cm diameter, 8-cm height, plugged with gauze) and the tubes were transferred to the ice surface. After 20–30 s, these wasps became inactive and then they were taken out from tubes to be conducted for the further tests. Next, 1-μL insecticide solutions were topically applied to the tergum using a microsyringe (Sangon Biotech, Shanghai, China). Wasps treated with 1 μL of acetone were used as controls. Post-treatment, all live parasitoid wasps were reared separately in the previously described above conditions and fed a 10% (*w*/*w*) honey solution. The survivors were collected for further analyses. Ten female adults constituted one biological sample and three replicates were tested.

### 2.5. qRT-PCR Validation

Total RNA was extracted using RNAiso Plus reagent (Takara Biotechnology Co. Ltd., Dalian, China), in accordance with the manufacturer’s protocol, from the whole bodies of surviving wasps after the 24-h insecticide exposure above mentioned. Approximately five survivors were pooled to prepare one sample. RNA quality and concentration were determined using a Nanodrop 2000 spectrometer (Thermo Fisher Scientific, New York, NY, USA). Each RNA sample was reverse transcribed using a PrimeScript RT reagent kit with gDNA Eraser (Takara, China) and then diluted to 70 ng/L with RNase-free H_2_O. qRT-PCR was performed on a QuantStudio™ Real-Time PCR system (Applied Biosystems, Foster City, CA, USA) using SYBR Premix Ex Taq II (Tli RNaseH Plus; Takara Biotechnology Co. Ltd., Dalian, China), in accordance with the manufacturer’s instructions. Each reaction (20 μL volume) was constituted by 10 μL of SYBR Premix Ex Taq II, 0.4 μL (10 μM) of each primer (Supplementary Table S1), 1.5 μL (75 ng) of cDNA template, 0.4 μL (50×) of ROX reference dye, and 7.3 μL of RNase-free H_2_O. The reaction program parameters were as follows: 95 °C for 5 min and 45 cycles of 95 °C for 15 s and 60 °C for 31 s. A no-template control and a no-reverse transcriptase control were both included on each reaction plate to test the underlying contamination. Reactions for all samples were independently repeated three times. *M. pulchricornis* β-actin (Acc: AQN78522.1) was chosen as the internal reference gene to normalize the target gene expression. The relative expression levels of *MpulCYP* genes among the different samples were measured using the 2^−ΔΔCt^ method [18]. Differences in the expression level of each target gene between the treatments were compared by one-way analysis of variance (Systat, Inc., Evanston, IL, USA) with Tukey’s post hoc test (*p* < 0.05) using R version 3.4.0 [19]. All the data were tested for the normality and homogeneity of variance and log or square root transformations were necessary to satisfy the hypothesis of normality and homogeneity of variance.

### 2.6. dsRNA Synthesis and Injection RNA Interference

RNA interference (RNAi) was conducted to further analyze the functions of *P450s* in *M. pulchricornis*. Gene-specific primers for dsRNA synthesis were designed with BLOCK-iT™ RNAi Designer (https://rnaidesigner.thermofisher.com/, accessed on 10 January 2021). The single-stranded Oligos of genes were synthesized following the manufacturer’s instructions and are listed in Supplementary Table S2. dsRNA was synthesized using an In Vitro Transcription T7 Kit (for siRNA synthesis) (Takara Biotechnology Co. Ltd., Dalian, China), and the concentration purity of dsRNA was assessed by a NanoDrop 2000 spectrophotometer. The dsRNA quality was further examined by 2% agarose gel electrophoresis, and the dsRNA was subsequently stored at −20 °C until use. Before dsRNA injection, tested wasps were kept in the glass tubes and these tubes were transferred to the ice surface for 20–30 s as mentioned above and then dsRNA was injected. Next, 0.5 μL dsRNA (50 ng/individual) was injected into the 2-day-old adults using a Nanoject II (Drummond Scientific, Broomall, PA, USA) with a microglass needle (0.53-mm inner diameter, 1.14-mm external diameter). After dsRNA injection, these wasps were collected and reared in a glass tube (2.2-cm diameter, 8-cm height, plugged with gauze) separately by feeding a 10% (*w*/*w*) honey solution. Total RNA was extracted after 12, 24, and 48 h to check the efficiency of RNAi by qRT-PCR. The dsRNA of the green fluorescent protein gene (GFP) was also synthesized as a negative control. *M. pulchricornis* adults were individually treated with 1 μL of the insecticide solution after 12 h of injection, and the method was the same as above. The death of individual wasps was recorded daily, and mortality rates were calculated as the mortality after 24 h of treatment. Each replicate included 10 wasps and was replicated three times.

## 3. Results

### 3.1. Identification of P450s and Phylogenetic Analyses

**Figure 1:**
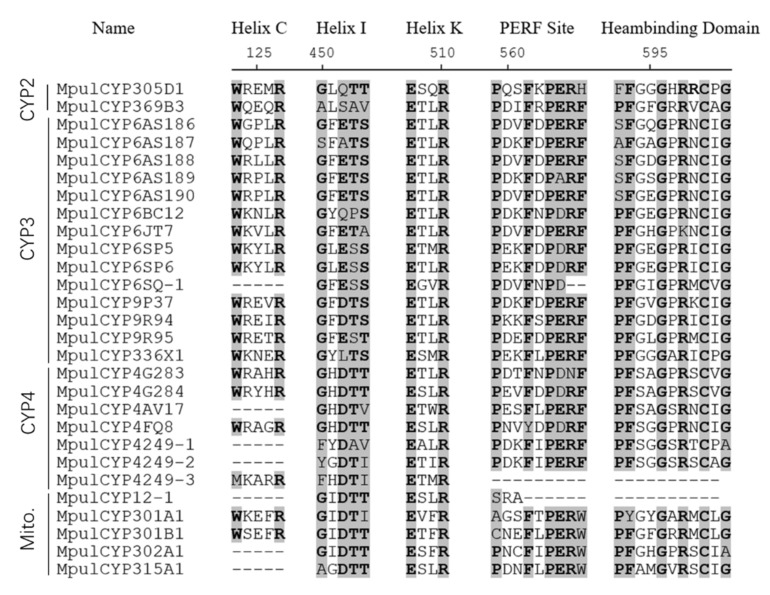
Alignment of functional domains of the 28 *MpulCYPs*. Conserved motifs are indicated in shadows.

A total of 28 P450 genes in *M. pulchricornis* (Table 1) were identified. Sequences of CYPs identified here were deposited in GenBank, and the accession numbers are listed in Table 1. All 28 genes contained full-length ORFs. The length of deduced *MpulCYP* proteins ranged from 209 to 557 amino acid residues. The theoretical pI ranged from 5.77 to 9.31, and the predicted molecular weight ranged from 23.92 to 116.83 kD. The BLASTx results showed that there were 22 P450 genes of *M. pulchricornis* which shared high sequence identity with P450s in *M. demolitor*. The results of conserved domain analyses revealed that 26 P450 genes had haem-binding motifs, 27 had PERF motifs, 21 had helix-C motifs, and all genes had helix-K and helix-I motifs (Figure 1).

**Figure 2:**
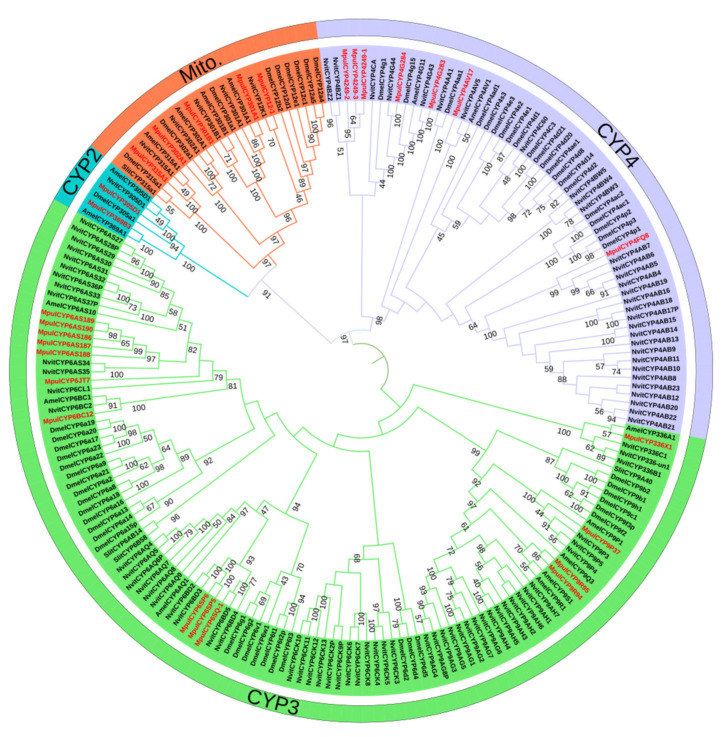
Phylogenetic analysis of P450s. *Meteorus pulchricornis* (Mpul), *Spodoptera litura* (Slit), *Apis mellifera* (Amel), *Drosophila melanogaster* (Dmel), *Nasonia vitripennis* (Nvit). Different colors indicate *CYPs* in different clans.

A neighbor-joining tree was constructed using the protein sequences of 28 MpulCYPs and P450s from four other insect species, namely *S. litura*, *A. mellifera*, *D. melanogaster,* and *N. vitripennis* (Figure 2). Of these, the CYP2, CYP3, CYP4, and mito clans contained 2, 13, 7, and 6 genes, respectively. The CYP2 and mito clans had fewer P450 genes compared with the CYP3 and CYP4 clans. There were two clusters, CYP305 and CYP369, in the CYP2 clan. In the CYP3 clan, the two largest clusters were CYP6 and CYP9. In the CYP4 clan, most genes were from the CYP4 cluster, and in the mito clan, the clusters included CYP12, CYP301, CYP302, and CYP315. Phylogenetic analysis revealed a close relationship between P450 genes from *M. pulchricornis* and *N. vitripennis*. Some P450 genes were orthologs, including *MpulCYP6AS187* and *NvitCYP6AS34* in the CYP3 clan, *MpulCYP4FQ8* and *NvitCYP4AB7* in the CYP4 clan, and *MpulCYP301B1* and *NvitCYP301B1* in the mito clan.

### 3.2. Relative Expression Profiling of P450s after Phoxim, Cypermethrin and Chlorfenapyr Treatment

**Figure 3:**
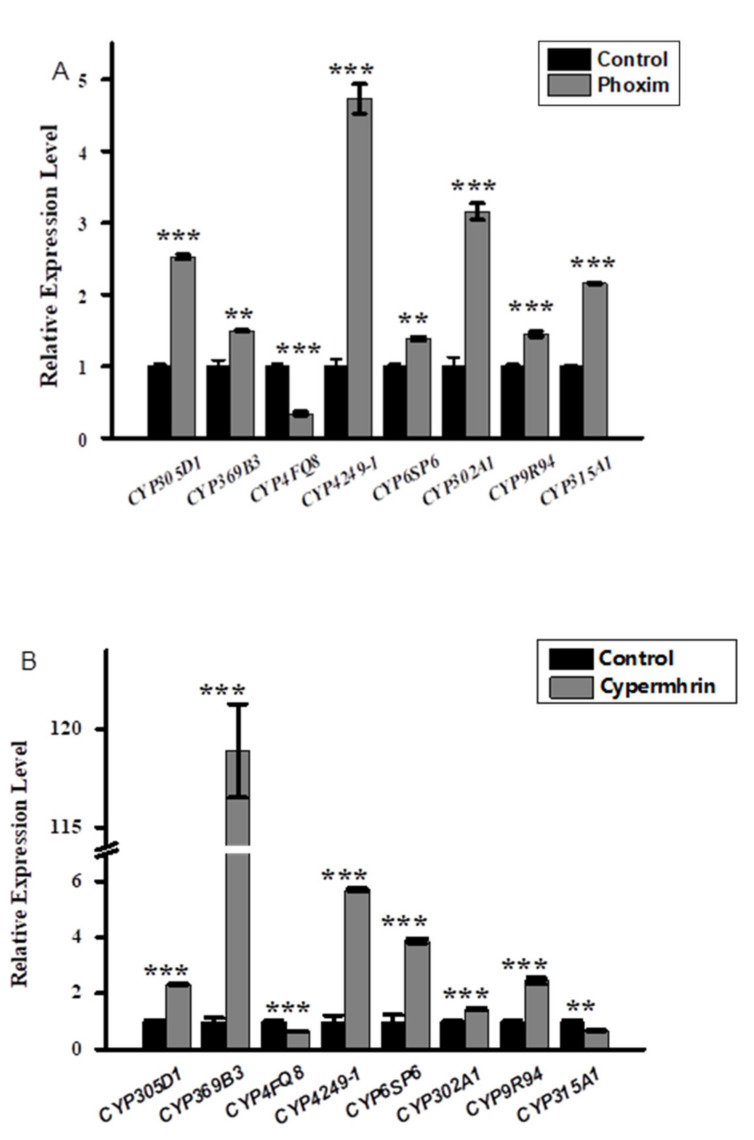

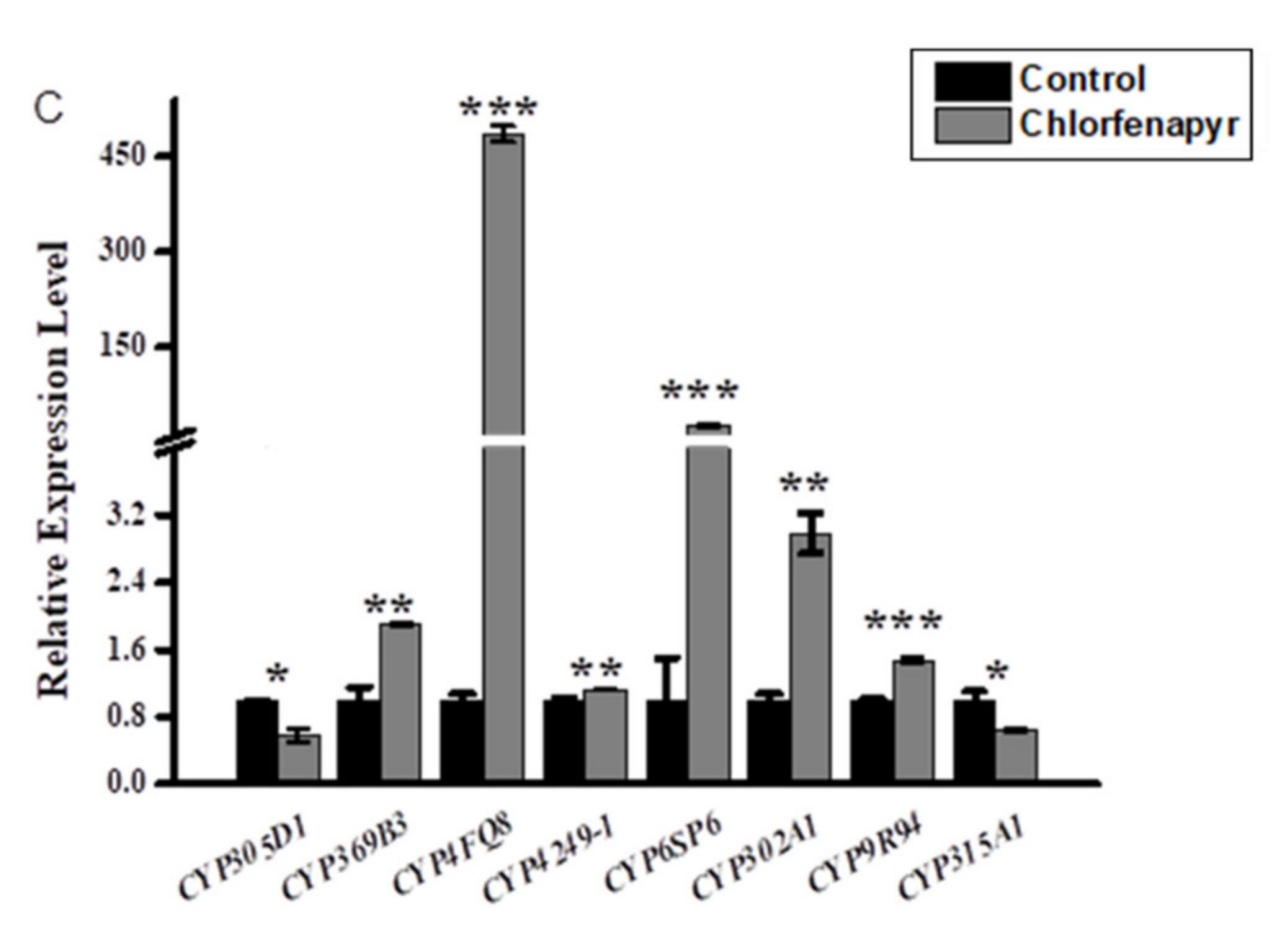
Relative expression profiles of *MpulCYPs* after phoxim (**A**), cypermethrin (**B**), and chlorfenapyr (**C**) treatment. The transcriptional level of each gene in insecticide-treated individuals was normalized relative to that in acetone-treated (control) individuals. Asterisks indicates a significant difference in relative expression levels (one-way analysis of variance with Tukey’s post hoc test, * *p* < 0.05; ** *p* < 0.01; *** *p* < 0.001). Log transformation was applied to satisfy the hypothesis of normality.

Three insecticides, namely phoxim, cypermethrin, and chlorfenapyr, were chosen to quantitatively measure the expression levels of the eight candidate *MpulCYPs* from four CYP clans by qRT-PCR (Figure 3). In response to phoxim exposure, the expression levels of seven *MpulCYPs* (*MpulCYP305D1, MpulCYP369B3, MpulCYP4249-1, MpulCYP6SP6, MpulCYP302A1, CYP9R94,* and *MpulCYP315A1*) were upregulated by 1.39–4.72-fold compared to the acetone control. The expression levels of *MpulCYP4FQ8* were downregulated by 66%. In response to cypermethrin exposure, the expression levels of six *MpulCYPs* (*MpulCYP305D1, MpulCYP369B3, MpulCYP4249-1, MpulCYP6SP6, MpulCYP302A1,* and *CYP9R94*) were upregulated by 1.4–118.89-fold compared to the acetone control. Remarkably, the expression levels of *MpulCYP369B3* were upregulated by 118.89-fold. The expression levels of *MpulCYP4FQ8* and *MpulCYP315A1* were reduced by 31% and 35%, respectively. In response to chlorfenapyr exposure, the expression levels of six *MpulCYPs* (*MpulCYP369B3, MpulCYP4FQ8, MpulCYP4249-1, MpulCYP6SP6, MpulCYP302A1,* and *CYP9R94*) were upregulated by 1.13–486.91-fold compared to the control. The expression levels of two *MpulCYPs* (*MpulCYP305D1* and *MpulCYP315A1*) were reduced by 36% and 42%, respectively. The expression levels of *MpulCYP4FQ8* were upregulated 486.91-fold. It is noted that *MpulCYP369B3, MpulCYP4249-1, MpulCYP6SP6, MpulCYP302A1,* and *CYP9R94* were upregulated by phoxim, cypermethrin, and chlorfenapyr exposure simultaneously.

### 3.3. RNAi for CYP369B3

**Figure 4:**
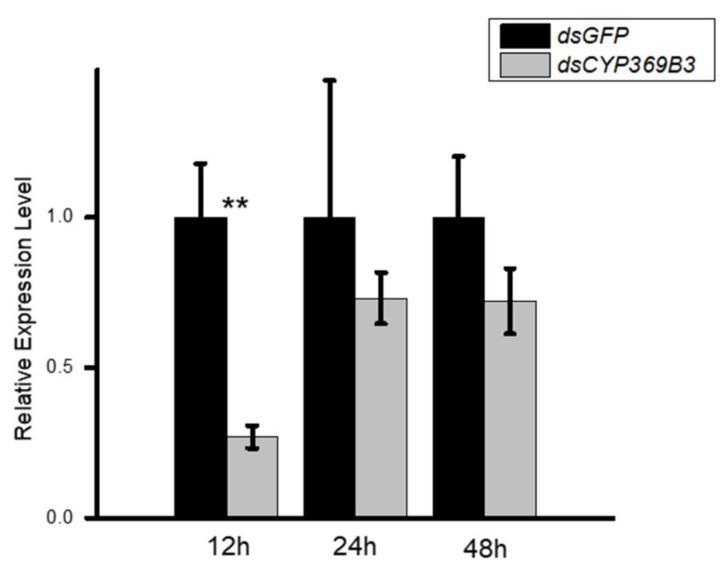
The analysis of the expression of *CYP369B3* after RNAi. Differences in the expression levels of each target were compared using a *t*-test. Significant differences are indicated by asterisks (** *p* < 0.01).

**Figure 5:**
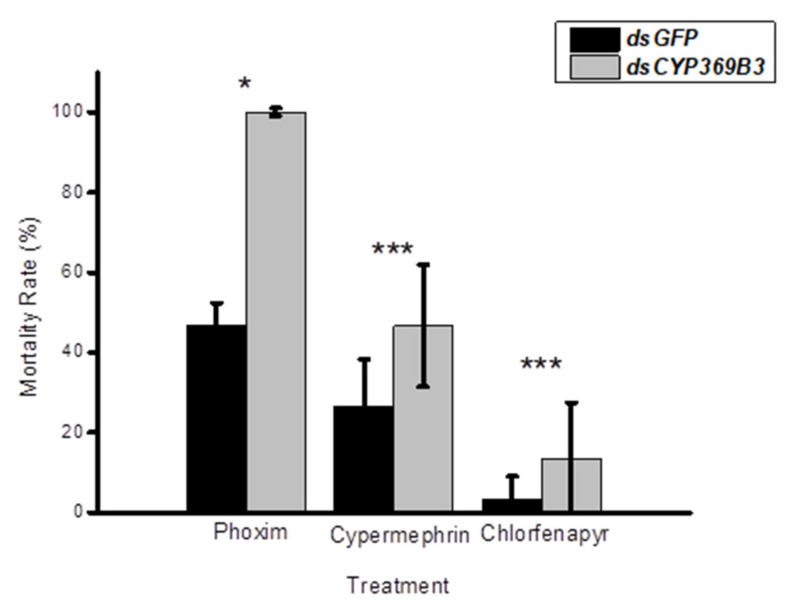
Effects of knocking down *CYP369B3* on the mortality of *Meteorus pulchricornis* adults after treatment with insecticides. (* *p* < 0.05; *** *p* < 0.001). Square root transformation was applied to satisfy the hypothesis of normality.

To investigate the roles of *MpulCYPs* in insecticides detoxification, *CYP369B3* was selected and successfully silenced by RNAi in 2-day-old adult wasps. The results showed that RNAi treatment significantly decreased the expression levels of *CYP369B3* 12 h after dsRNA injection. Although the expression levels of *CYP369B3* were decreased 24 and 48 h after silencing, there were no significant differences between dsRNA and dsGFP injection groups (Figure 4). Furthermore, after *dsCYP369B3* injection, the mortality adult was increased by 114.27%, 74.39%, and 300.30% compared to *dsGFP* injection groups when they exposed to phoxim, cypermethrin, and chlorfenapyr, respectively (Figure 5).

## 4. Discussion

The extensive application of insecticides caused significant negative effects on the natural enemies of pests [20]. Thus far, many insecticides have been found to have strong toxicity to parasitic wasps. For instance, phoxim and cypermethrin were reported to be harmful to *M. pulchricornis* [16]. Yang et al. [21] demonstrated that chlorpyrifos, chlorantraniliprole, emamectin benzoate, and spinosad were toxic to *Trichogramma japonicum* and *Trichogramma dendrolimi*. Although the lethal and/or sublethal effects of insecticides on parasitoid wasps have been tested in a wide range of insecticides, the detoxification mechanisms of parasitoid wasps have received less attention.

P450s have been previously identified in many kinds of insects, and these CYPs were suggested to participate in various physiological processes, such as detoxication [22], pesticide resistance [23,24], immune response [25], and cuticularization [26] in insects. To date, studies regarding the detoxication functions of P450s have mainly focused on agricultural or forestry pests, but there is very limited research on non-target insects, especially on parasitoid wasps.

Identification of P450 genes is a key step in investigating their roles in insects. In this study, 28 CYPs were identified from the transcriptome of *M. pulchricornis.* The number of P450 genes found in *M. pulchricornis* differed significantly from the number of CYP genes found in other wasps, such as *A. mellifera* (50 *CYPs*; [27]), *N. vitripennis* (92 *CYPs*; [28]), *Macrocentrus cingulum* (41 CYPs), *Microplitis demolitor* (47 *CYPs*), and *Ceratosolen solmsi* (39 *C*YPs; [29]. This is mainly due to the diverse functions of P450s among insect species [30]. Furthermore, the depth of sequencing may also affect the number of P450s screened here.

It is well known that *MpulCYPs* are important players in the regulation of the degradation of insecticides in insects. CYP2 clans of P450s are often involved in environmental response, and mito clans are involved in the synthesis of ecdysone [31,32]. In contrast, CYP3 and CYP4 are well known for their multiple roles in ecological adaptation in insects, such as the metabolism of exogenous toxic chemicals from the environment or hosts, participation in fatty acid hydroxylation, and biosynthesis [33]. Therefore, the number of *CYP3* and *CYP4* clans identified from *M. pulchricornis* was dramatically higher than that of the Mito and CYP2 clans.

Phylogenetic analysis revealed a close relationship between P450 genes from *M. pulchricornis, A. mellifera*, *D. melanogaster,* and *N. vitripennis*. The homology between AmelCYP4G11 and MpulCYP4G283 was 100%, and AmelCYP4G11 may play an important role in the hygiene behavior and antioxidant process of *A. mellifera* [34]. Therefore, it was speculated that *MpulCYP4G283* may play the same role in *M. pulchricornis*. The homology of *MpulCYP6SP6*, *MpulCYP6SP6,* and *MpulCYP6SQ-1* was 100%, and the homology of these three genes to *DmelCYP6G1* was 94%. Overexpression of *DmelCYP6G1* in *D. melanogaster* contributed to the metabolic resistance to dichlorodiphenyltrichloroethane, imidacloprid, acetamiprid, nitenpyram, and malathion [35]. Therefore, it was speculated that these three genes played an important role in insecticide metabolism.

Cytochrome P450 has both a highly conserved core region (haem-binding site and oxygen-binding site) and a variable region associated with substrate recognition and substrate binding (substrate-binding site). The conserved cysteine (Cys) in the haem-binding motif (FxxGxxxCxG) is responsible for the absorption peak of the CO-binding protein at 450 nm. Helix-I is presumed to be the binding site of molecular oxygen, and helix-K may play a role in stabilizing the core structure of cytochrome P450 proteins [2]. In this study, 21 CYP protein sequences had conservative CYP domains. Studying the structure of different cytochrome P450 monooxygenases not only contributes to the further understanding of the relationship between the structure and function of P450, but also provides theoretical reference for the development of new insecticides based on the molecular structure of P450.

Studies have shown that the P450 oxidase system is one of the main functional enzymes in insecticide metabolism, which can improve the ability to degrade insecticides by increasing the P450 expression level. In the present study, to further investigate the potential role of P450s in the tolerance mechanism in *M. pulchricornis* when they suffered from commonly used insecticides in mulberry fields, the expression of P450s in *M. pulchricornis* was validated after phoxim, cypermethrin, and chlorfenapyr exposure. The results showed that most MpulCYPs significantly increased their expression levels after insecticide stress. In *Musca domestica*, *CYP6D1* was overexpressed in resistant strains of pyrethroid and organophosphorus and had a metabolic effect on insecticides [36]. Both *CYP6G1* in *D.* *melanogaster* and *CYP6AY1* in *Nilaparvata lugens* was upregulated and contributed to the metabolic effect on imidacloprid insecticides [37,38]. Regulating the expression of the P450 gene caused *T. vaporariorum* to develop imidacloprid resistance. Compared with susceptible strains, *CYP6ER1* in *N. lugens* was significantly overexpressed in the field-collected resistant strains, which further provided a basis for P450 to participate in the generation of insect resistance [39]. This further provides a strong basis for demonstrating that P450 is involved in the development of insect resistance.

In addition, P450 genes were not only extensively affected by insecticide stress, but also had the function of responding to multiple selection pressures from insecticides. Although the substrates of different P450 genes are different, they may overlap to adapt to environmental changes. The expression of several P450 genes in response to the induction of exogenous compounds, such as insecticides, may also be used to respond to the selection pressure of insecticides, showing evolutionary plasticity [40]. The expression of 17 P450 genes was significantly upregulated by chlorantraniliprole and fipronil stress in *Mythimna separata* [41]. Similarly, in the present study, almost all the selected P450s genes in *M. pulchricornis* were upregulated and the expression levels of *MpulCYP369B3, MpulCYP4249-1, MpulCYP6SP6, MpulCYP302A1,* and *CYP9R94* were significantly increased after exposure to phoxim, cypermethrin, and chlorfenapyr. Therefore, it can be speculated that these P450s genes were involved in the detoxification of phoxim, cypermethrin, and chlorfenapyr.

RNAi technology has been widely used to uncover the functions of genes involved in insect detoxification. Sun et al. [42] evaluated the role of *CYP6FU1*, *CYP425A1,* and *CYP6AY1* in the resistance of *N. lugens* to etofenprox using RNAi and they found that knocking down these genes resulted in the higher sensitivity to etofenprox. Silencing of the CYP6BG1 gene in *Plutella xylostella* demonstrated its role in chlorantraniliprole resistance [43]. Using RNAi, Mao et al. [44] evaluated the impacts of *CYP6ER1*, *CYP302A1*, and *CYP3115A1* in nitenpyram resistance in *N. lugens*. When the three P450 genes were successfully silenced, the sensitivity of *N. lugens* to nitenpyram was increased. Similarly, the *CYP6ER1* gene was demonstrated to involve in the resistance of *N. lugens* to thiamethoxam and dinotefuran, similar to imidacloprid, and the knockdown of *CYP6ER1* dramatically increased the toxicity of clothianidin to *N. lugens* [45]. In this study, *MpulCYP369B3* was significantly upregulated by the three insecticides, and expression was upregulated 118-fold after cypermethrin stress. When *MpulCYP369B3* was successfully knocked down, the mortality of *M. pulchricornis* significantly increased. Therefore, *MpulCYP369B3* may play an important role in response to insecticide stress in *M. pulchricornis*.

In summary, the present study reported the large-scale identification of P450 genes in *M. pulchricornis*, and their expression patterns were significantly affected by insecticide treatment. Knocking down *CYP369B3* resulted in increased mortality in adult females after insecticide exposure. The results confirmed the vital roles of P450s in parasitoid wasps when they were subjected to insecticides. The present study can lay a foundation for an in-depth understanding of the function of P450s in parasitoid wasps.

## Figures and Tables

**Table 1:** Sequence information of identified *MpulCYP* genes.

Gene Name	Acc. No	Encoded Protein Length/aa	Molecular Weight/kD	Isoelectric Point	Blastx Best Hit				
					Species	Protein Name	E Value	Identity %	Accession
*MpulCYP4G283*	MZ169337	557	63.23	7.65	*Fopius arisanus*	cytochrome P450 4g15	0	89.61	XP_011304723.1
*MpulCYP4G284*	MZ169336	555	63.44	6.57	*Microplitis demolitor*	cytochrome P450 4g15	0	80.29	XP_014300333.1
*MpulCYP4AV17*	MZ169338	313	35.86	6	*Microplitis demolitor*	cytochrome P450 4c3-like	1.00 × 10^−155^	69.01	XP_008555432.1
*MpulCYP4FQ8*	MZ169339	518	59.79	8.61	*Microplitis demolitor*	cytochrome P450 4C1	0	69.56	XP_008550233.1
*MpulCYP6AS186*	MZ169329	504	58.35	9.18	*Microplitis demolitor*	probable cytochrome P450 6a14	0	59.84	XP_008560120.1
*MpulCYP6AS187*	MZ169330	500	57.2	9.25	*Microplitis demolitor*	probable cytochrome P450 6a14	0	56.10	XP_008560120.1
*MpulCYP6AS188*	MZ169333	500	57.59	9.15	*Microplitis demolitor*	cytochrome P450 6k1	0	57.75	XP_008557726.1
*MpulCYP6AS189*	MZ169331	499	57.42	8.28	*Microplitis demolitor*	probable cytochrome P450 6a14	0	67.34	XP_008560120.1
*MpulCYP6AS190*	MZ169332	502	58.13	8.49	*Microplitis demolitor*	probable cytochrome P450 6a14	0	68.76	XP_008560120.1
*MpulCYP6BC12*	MZ169334	522	60.41	8.85	*Polistes dominula*	cytochrome P450 6B5-like	0	65.31	XP_015174482.1
*MpulCYP6JT7*	MZ169328	498	56.83	6.26	*Microplitis demolitor*	cytochrome P450 6a2-like	0	61.04	XP_014299556.1
*MpulCYP6SP5*	MZ169326	512	58.99	9.18	*Diachasma alloeum*	cytochrome P450 6k1	0	61.05	XP_015121128.1
*MpulCYP6SP6*	MZ169327	502	58.3	9.31	*Diachasma alloeum*	cytochrome P450 6k1	0	57.49	XP_015121128.1
*MpulCYP6SQ-1*	MZ169325	268	30.38	6.13	*Orussus abietinus*	cytochrome P450 6k1-like	3.00 × 10^−139^	70.63	XP_012272474.2
*MpulCYP9P37*	MZ169322	505	58.95	7.58	*Belonocnema treatae*	cytochrome P450 9e2-like	0	58.16	XP_033219303.1
*MpulCYP9R94*	MZ169321	508	58.7	7.97	*Microplitis demolitor*	cytochrome P450 9e2-like	0	61.55	XP_008557951.2
*MpulCYP9R95*	MZ169323	507	58.28	6.9	*Microplitis demolitor*	cytochrome P450 9e2-like	0	58.65	XP_008557951.2
*MpulCYP12-1*	MZ169346	224	25.57	9.16	*Microplitis demolitor*	cytochrome P450 CYP12A2	8.00 × 10^−96^	71.14	XP_008548500.1
*MpulCYP301A1*	MZ169345	525	60.42	8.48	*Microplitis demolitor*	probable cytochrome P450 301a1, mitochondrial	0	83.69	XP_008548437.1
*MpulCYP301B1*	MZ169342	513	60	8.8	*Microplitis demolitor*	probable cytochrome P450 49a1	0	76.26	XP_014298339.1
*MpulCYP302A1*	MZ169344	302	34.26	8.19	*Microplitis demolitor*	cytochrome P450 302a1, mitochondrial	1.00 × 10^−141^	66.01	XP_008552208.1
*MpulCYP305D1*	MZ169319	492	56.32	8.58	*Microplitis demolitor*	probable cytochrome P450 305a1	0	69.56	XP_014295888.1
*MpulCYP315A1*	MZ169343	222	25.09	7.71	*Microplitis demolitor*	cytochrome P450 315a1, mitochondrial	9.00 × 10^−92^	0.5936	XP_008559430.1
*MpulCYP336X1*	MZ169324	504	58.01	8.55	*Microplitis demolitor*	cytochrome P450 9e2	0	0.5029	XP_008557820.1
*MpulCYP369B3*	MZ169320	505	116.83	6.75	*Microplitis demolitor*	probable cytochrome P450 304a1	0	59.12	XP_008561134.1
*MpulCYP4249-1*	MZ169335	258	29.53	8.64	*Microplitis demolitor*	cytochrome P450 4C1-like	9.00 × 10^−105^	57.60	XP_014298773.1
*MpulCYP4249-2*	MZ169340	209	23.92	8.91	*Microplitis demolitor*	cytochrome P450 4C1-like	6.00 × 10^−73^	52.48	XP_014298773.1
*MpulCYP4249-3*	MZ169341	301	34.15	5.77	*Microplitis demolitor*	cytochrome P450 4C1-like	4.00 × 10^−92^	48.44	XP_014298773.1

## Supplementary Materials

The following are available online at https://www.mdpi.com/article/10.3390/insects12070651/s1, Table S1: Primers used for real-time quantitative PCR, Table S2: Primers used for RNA interference.
