# Supplementary material for: Cytochrome P450s Are Essential for Insecticide Tolerance in the Endoparasitoid Wasp Meteorus pulchricornis (Hymenoptera: Braconidae)
Source: Insects. 2021 Jul 16;12(7):651. doi: 10.3390/insects12070651 (PMC8306486; doi:10.3390/insects12070651)
Supplement: Supplementary file 1 [file insects-12-00651-s001.zip › insects-1270277-supplementary.pdf]

Table S1 Primers used for real-time quantitative PCR

Gene	Forward primer	Reverse primer	Product Size (bp)
CYP305d1	TCTGGAGTCTTGCAACTGGC	ATGACTCCGGTGCAATGTGT	150
CYP304a1	TTTATCCAAACCGTGCCCGT	GGCGACCGAATCCGAAATTG	123
CYP4c4	TGACCTTCTCATTGCCGCTT	GCTCGTGACTGTATGCCCTT	162
CYP4c1	CCATTCAGTGGTGGTTCACG	TCCGTCCAAGGACCGAATTG	162
CYP6b3	GGACCTCGCATTTGCATTGG	TTCCATTGACGTCGGGTGTT	154
CYP302a1	CGGTTGCGGTGACACAAAAT	GGACCGTGACCGAATGGTAA	157
CYP9a1	TTCGTTGATCCAGTTGCGGA	GCTTGACGTAAATGCCGGAC	108
CYP315a1	GAAAGGTCTTGAGGGGGACG	AGCGGCCAAGATAAGATCCG	100
β-actin	ACCTGAAGAACATCCCGTCCTTT	ACGACCAGAGGCATAAAGGGAAA	148

Table S2 Primers used for RNA interference

Gene	*dsCYP369B3*	*dsGFP*
Oligo 1	GATCACTAATACGACTCACTATAGGGCCTCACAACACAATTCATTTT	GATCACTAATACGACTCACTATAGGGGGGATGTCTCACATCTTGTTT
Oligo 2	AAAATGAATTGTGTTGTGAGGCCCTATAGTGAGTCGTATTAGTGATC	AAACAAGATGTGAGACATCCCCCCTATAGTGAGTCGTATTAGTGATC
Oligo 3	AACCTCACAACACAATTCATTCCCTATAGTGAGTCGTATTAGTGATC	AAGGGATGTCTCACATCTTGTCCCTATAGTGAGTCGTATTAGTGATC
Oligo 4	GATCACTAATACGACTCACTATAGGGAATGAATTGTGTTGTGAGGTT	GATCACTAATACGACTCACTATAGGGACAAGATGTGAGACATCCCTT
